# Humanistic Approach to Nursing Education: Lived Experiences of Iranian Nursing Students

**DOI:** 10.5539/gjhs.v7n2p87

**Published:** 2014-09-28

**Authors:** Shahrzad Ghiyasvandian, Fariba Bolourchifard, Zohreh Parsa Yekta

**Affiliations:** 1 School of Nursing and Midwifery, Tehran University of Medical Sciences, Tehran, Iran; 2 School of Nursing and Midwifery, Shahid Beheshti University of Medical Sciences, Tehran, Iran

**Keywords:** academic nurse teacher, nursing education, student, hermeneutic phenomenology

## Abstract

The nurse teachers tried to have a complete understanding of the educational contents, to transfer knowledge to nursing students better, and to facilitate the process of education. The purpose of this study was to explore the lived experiences of Iranian nursing students regarding the characteristics of academic nurse teachers. In this hermeneutic phenomenological study, data were collected via in-depth, semi-structured interviews with 12 Iranian nursing students and the audio-taped and transcribed interviews analyzed according to Van Manen´s method. The main theme emerged during data analysis, was “humanistic approach to nursing education”. The theme was extracted from 2 sub-themes including ‘ethical necessities’ and ‘effective interaction’. The findings present greater understanding of humanistic approach to nursing education.

## 1. Introduction

Education consists of basic efforts to empower individuals with knowledge in order to prepare them for future and to provide an infrastructure for lifestyle in human society. Also, higher education institutes and universities are responsible for different roles and tasks, but most experts have stated three primary roles for universities that include education, research, and services (Sahenk, 2010). In addition, university is considered as one of the most important environments for learning and gaining education. Also, universities play a significant role in providing training services, which suits the expectations of society and consequently in producing educational services. In this regard, teachers play a specific role in students’ learning and obtaining academic achievements. Effective learning is importantly dependent on the teachers and their actions in the classroom (Dunlosky et al., 2013).

Teachers are greatly important in process of teaching–learning activities. Teachers should be able to educate capable workforce in all aspects of knowledge, attitude, and skill. To reach this aim, they must have some criteria to be able to meet systematic (university) and meta-systematic (society) expectations, as well. Thus, each of the meta-system and system stakeholders has expectations. In addition, they provide meeting their expectations through a well-defined set of criteria. Researchers show that teachers should have the highest standards in education, research and management (Srisawasdi, 2012; Grierson, 2010; Kelly, 2007). Similarly, one of the most basic and the most important beneficiaries of teachers are students who have expectations of the faculty with determined criteria. In addition to knowledge, the teacher’s personality, as well as their behavior is important for students because experiencing the proper pattern of behavior is an incentive for educating students. Furthermore, student– teacher communication affects students’ attitudes (Vaismoradi & Parsa-Yekta, 2011). Researches have reported different views on the characteristics of a good teacher such as teaching skills, teacher–student communication and teacher’s characteristics (Sahenk, 2010; Grierson, 2010). A study has been conducted on the characteristics of a good teacher which pointed out teacher’s mastery over course content, their general knowledge and their teaching skills (Hossein et al., 2010).

It seems that the demands and expectations of nursing students from academic nurse teachers are changing with regard to educational condition. Thus, obtaining lived experiences of them may lead to developing considerable data.

### 1.1 Background on Nursing Education in Iran

Nursing education is the basic course of medical sciences education in Iran (Jahanpour et al., 2010). In Iran undergraduate nursing students should pass 135 units including 26 basic sciences course units, 20 general course units, and 89 specialized course units for getting bachelor’s degree (Borhani et al., 2010). In this regard, most of the Iranian nurse teachers have a Master’s degree or PhD in nursing (Emamzadeh Ghasemi et al., 2014). Also, the role of Iranian nurse teachers has been highlighted as his/her effectiveness is thought to be dependent on the ability to have an effective relationship with nursing students. The close relationship between nurse teacher and student may affect the quality of nursing education (Vaismoradi & Parsa-Yekta, 2011), and active involvement of both teacher and student is necessary for effective nursing education in Iran (Hossein et al., 2010).

### 1.2 Aim

The purpose of this research was to understand the lived experiences of Iranian nursing students about the characteristics of academic nurse teachers.

## 2. Method

Our research was a hermeneutic phenomenological methodology of Van Manen (2001). It was focused on lived experience of nursing students to uncover the meaning of the characteristics of academic nurse teachers as experienced by the individuals via their life stories. The corresponding author who is an academic nurse educator presented deep questions to make sense of the experience about the subject. Then authors searched the literature about the lived experiences of persons to guide participants. We used thematic analysis to clarify the experience of characteristics of academic nurse teachers. An exhaustive description of the experience of characteristics of academic nurse teachers was found. Then researchers tried to understand the experience of characteristics of academic nurse teachers in a more humanitarian sense. Authors concurrently considered the total and the contextual data to understand the contribution of each part to the formation of the phenomenon of our study.

### 2.1 Participants and Data Collection

Due to the sensitive nature of the phenomenon under the study, a purposive sampling technique was implemented to recruit 12 participants from Nursing School of Tehran University of Medical Sciences. Participants were selected on the basis of the maximum variation including age, gender and years of experience as nursing student and have agreed to participate in the study. Average age of them was 20.2 (SD = 2.1). Seven of participants were female. The main criterion for inclusion was the experience of the characteristics of academic nurse teachers. All of participants were recruited from bachelors’ degrees, and had studied nursing for 1-4 years.

In this study, data were collected through in-depth, semi-structured interviews by the corresponding author. The participants were asked to talk about their experiences of characteristics of academic nurse teachers. The starting questions were “what is the meaning of the characteristics of academic nurse teachers?” Probing questions were also used to clarify participant’s descriptions. Nursing students were interviewed in a safe and private place by the researcher. Interviews lasted between 45 to 60 minutes. Three of participants were interviewed during 2 sessions; other participants were interviewed during 1 session. All interviews were tape-recorded and transcribed verbatim in the Persian and then the transcribed interviews and field notes were coded. The interviews that were related to the aim of this study were interpreted to English by an expert interpreter and then the English was interpreted to Persian for confirmation by an Iranian bilingual translator. Data collection was continued until no new themes were being generated.

### 2.2 Data Analysis

Data analysis was begun simultaneously with data collection. The data were analyzed using van Manen’s holistic, selective and detailed approaches (Van Manen, 2001). In the holistic approach, each transcript was read as a whole to gain an understanding of the texts in general. This is followed by the selective approach in which each transcript was read several times, looking for phrases from the participants that appeared particularly essential about the studied phenomenon, the experience of characteristics of academic nurse teachers. Then, these phrases were highlighted to identify themes from the early concepts. Finally, in detailed approach, author went back to the transcripts, examining each sentence for meaning units. The formulated meanings were interpreted and the subthemes were identified. Then, the sub-themes were clustered into the main theme. The sub-themes were reviewed and revised with research team members and were integrated into a comprehensive phenomenon.

### 2.3 Methodological Considerations

The trustworthiness of this study is evaluated (Streubert & Carpenter, 2010). Credibility was established through prolonged engagement with participants and extended immersion. Member checking and peer reviewing were done to verify the findings. Triangulation of data collection methods was also done as field note writing. Transferability was enhanced by purposive sampling of nursing students who had experienced characteristics of a good nurse teacher. Detailed description of results as well as returning to literature when interpreting the data was done to support the results. An extensive audit trail was created to ensure confirmability.

### 2.4 Ethical Consideration

The ethics committee of Tehran University of Medical Sciences approved the study. The data collection was carried out after obtaining verbal consent and a signed informed consent form from the participants. The students were given verbal and written information about the study. They had the right to withdraw from the study at any time during or after the interviews and could ask the researchers to return their audio-taped interviews. The interview setting was a quiet location in the nursing school, on the basis of participant convenience and preference.

## 3. Findings

In this study, the lived experiences of nursing students showed that the main theme of “humanistic approach to nursing education” was emerged of two sub-themes including the “ethical necessities” and “effective interaction”.

### 3.1 Ethical Necessities

One important aspect of humanistic approach to nursing education about a good nurse teacher was the ethical necessities. Our participants emphasized the importance of ethical aspects in nursing education and mentioned a teacher’s morality as one of the characteristics of academic nurse teachers. Considering ethics in responding nursing students’ questions, speaking nicely, avoiding wasting nursing students’ time, and respecting nursing students’ personality are examples of ethical aspects in nursing education. This is evident in the statements of the students:

“*Classes should be stress-free to make it possible for me as a nursing student to talk without stress and simply ask my question. It often happens that you don’t dare to ask a question. When you ask a question, the nurse teacher makes fun of you: ‘Is your knowledge that little to ask a question like this?’*.” (participant5).

Another factor that was stated by nursing students was nurse teacher’s attention to questions and responding them correctly. In fact, nursing students expect a good teacher not to make fun of their questions and answer them with respect. Also, they emphasized that not responding students’ questions are an aspect of disrespecting. To them, an Academic Nurse Teacher is the one who develops confidence followed by creativity in nursing students. One of the participants stated:

“*When we asked a question she humiliated us in front of others, she humiliated us in front of each other, and this was too bad*.” (p8).

A participant stated:

“*We had a teacher who valued her class so much. Well, some teachers didn’t even come to class after passing half an hour of class time. When the teacher doesn’t care regarding her class, how could he expect students to attend her class enthusiastically. That certain teacher always came on time, left on time and she was so strict about her class. She asked us to respect the class as she did and to respect the presence of one another as well*.” (p10).

Another nursing student said:

“*Some of our teachers are of high academic levels; they respect nursing students too much. For instance, when a nursing student comes in the class, she just stands up. Moreover, she is so strict regarding her meetings with nursing students. If she says she will come to a certain class at a certain time, she always comes on time*.” (p12).

In this respect, another nursing student said:

“*We had a teacher who was really perfect. Her teaching, her behaving with nursing students was great. She respected all of us and we respected her, too. I never forget her and always try to be just like her. She was a good nurse teacher for me*.” (p3).

### 3.2 Effective Interaction

Another aspect of humanistic approach to nursing education about a good nurse teacher was related to effective interaction. One of the characteristics, which were mentioned by nursing students several times as a characteristic of Academic Nurse Teachers, was effective interaction with nursing students. In other words, while entering class, greeting nursing students, knowing them, responding their questions, and guiding them are indicators of interacting with nursing students and are good behavior of teachers. Here are some narrations about effective interaction stated by students in their lived experiences.

A nursing student said:

“*We have a teacher that all students like. She becomes a good nurse teacher because of her good interaction with nursing students, she talks to students and even answers the questions that are out of the subject lesson; she even talks to us individually*.”(p11).

Students participating in the study mentioned creating an intimate atmosphere, filled with vitality by good nurse teachers. In other words, they experienced that despite the need for a disciplined framework for class; a good nurse teacher should be flexible and should prevent from students’ boredom. Participants thought that engaging students in a classroom, in responding questions and giving opportunities for short breaks between classes help creating a happy atmosphere. Regarding the atmosphere of the class, one of participants said:

“*Her class was so boring and we weren’t allowed to even move or laugh. In my experience, a class needs flexibility as well as management which makes students relaxes in the class and not to think that there is an obligation which makes them reluctant… Class must be made in a way that students can participate. I mean, it should be in a way that we participate in the class, too*.” (p1).

Based on students’ experiences, an Academic Nurse Teacher is the one who is not too proud of herself and who is able to understand students. According to nursing students, teacher’s pride is a barrier to class flexibility and a factor that increases stress and anxiety in the class. They showed a proud teacher the one who does not understand students’ condition and distances herself from students. One nursing student narrated her lived experience in this regard:

“*I had a teacher that I really hated her course. I studied that lesson with too much stress, a lesson which was so easy but I sat in the class full of stress because the teacher was so arrogant*.” (p7).

## 4. Discussion

In this study, the humanistic approach to nursing education was indicated as the nursing students described their lived experiences. In this regard, this research provides the humanistic approach to nursing education as an essential aspect of experiences obtained by the Iranian nursing students regarding the characteristics of academic nurse teachers. The lived experiences of nursing students participating in this research suggest that the humanistic approach as one of the most important cases of characteristics of academic nurse teachers consisted of two sub-themes including “ethical necessities” and “effective interaction”.

Nursing students experienced tolerance and openness as ethical necessities of academic nurse teachers. In this regard, to some nursing students, observing some of the essence of the ethical is superior to any other characteristic. According to nursing students, respecting students’ personality by paying attention and understanding her/his, respecting time of the class and time of the nursing students and supporting them are ethical characteristics of academic nurse teachers. In fact, to nursing students, a teacher who is of high academic degree but does not consider nursing students, class, and request of them is not academic nurse teachers. Also, according to participants, the ethical characteristics of a nurse teacher play a specific role in making them academic nurse teachers in nursing education. The aim of Heidarzadeh et al. (2012) study was to find out the perspective of clinical teacher s and nursing students on the features of an efficient clinical teacher in nursing. It was a qualitative study based on content study. The view point of nursing students was only one theme, personal characteristic. According to the students the most personal characteristics were ethical and emotional personality and their supportive role. The result of this study identifies some individuality of efficient clinical teachers and can be used as a guideline in clinical administration and instruction. It is also suggested that more studies be done to support the application of these characteristics in clinical education. Our findings demonstrate ethical characteristics of academic nurse teachers that were expressed by nursing students such as dealing respectfully with students and avoid wasting their time. In present study, characteristics of a capable teacher for effective teaching indicated a mentioned characteristic by students as following: maintaining dignity and respect for students. In fact, ethical support has an important role in increasing academic achievements (Torrance et al., 2012). In addition, the teachers protect students from inappropriate professional and academic behaviors (Yunus et al., 2011). The study of the Zarghami and Ramezani (2012) compared the existence philosophers’ ideas about the nature of ethics and man’s ethical life as well as to infer ethical fundamentals and related principles of teacher-student relationships. The research methodology was comparative analysis (a qualitative research approach) and practical syllogism (a philosophical research method). In relation to the findings of this research “the existence thinkers lay emphasis on common points with regard to man’s existence and his ethical life. On this basis “three fundamentals and six principles are proposed for ethical relationships between teacher and student: the fundamental of validity of personality and unique characteristics of human being and the principles of respect to individuality and choosing student-teacher relations instead of teacher-student relations; the fundamental of validity of originality and the principles of choosing me-you relations instead of me-that relations with student by the teacher, establishment of being relations with student by the teacher and preparing the grounds by the teacher for experiencing border situations by the students; and finally “the fundamental of validity of emotional and exiting aspects of life on the side of rational aspect and the principle of providing information about human being and preparing the grounds for participation of student in the real life. Finally, referring the shortcomings of the ideas of these philosophers “it is concluded that on the side of following ethical fundamentals and principles in relations between teacher and student in the higher education system of our country “due attention should be paid to the shortcomings as well.

The results of our research highlight the importance of the role of nurse teachers in maintenance of ethical necessities in the all stage of nursing education. In this regard, the nursing students participating in this study stated the role of nurse teacher as a person promoting ethical necessities. The participants experienced that the individual’s characteristics such as flexibility, is very important in the process of nursing education.

In addition, our study showed that students considered flexibility of class atmosphere and avoiding harsh behaviors. In a study of Sahenk (2010) about the characteristics of an effective teacher in creating a good learning environment, it was demonstrated that communication associated with good behavior is of characteristics of a good teacher. A teacher who has a friendly behavior with nursing students and who applies positive relationship approaches and respects each student highly motivates students to participate. A friendly relationship and a good and supportive behavior with students enhances their motivation, their work in the classroom, increases criticism acceptance, better adaptation to stress and paying more attention to the educational content presented by the teacher (Smedley& Morey, 2010; Ramos et al., 2013). Mazloomi Mahmood abad et al. (2010) conducted their study to find out the characteristic of a professional faculty member based on opinion of medical students in Iran. It was an analytic-descriptive study. Total sample were 240 medical students. The results showed the most features of an expert faculty member were communication ability. They recommended improve communication skilled for faculty members. In fact, for building a good relationship between nurse teacher and student, teacher’s behavior, his ability to empathize and motivating students played important roles (Shahsavari et al., 2013). The study of Elahi et al. (2012) was to determine the nursing students’ perceptions and experiences regarding features of effective education (both theoretical and clinical) in Iran. This is a qualitative study. Three main themes were found as the factors involved in the effectiveness of education: Teacher’s level of competency and expertise. Development of students’ competence proficiency, and professional skill; and effective and valid evaluation. The participants believed these are the key factors to the development of capability among nursing students and enables them to provide more effective health care. In contrast, according to nursing students’ experiences, in this study, an academic nurse teacher who just enters the class just to teach the targeted content and leaves class without any interaction with students could not be a good teacher. However, the nurse teachers must grow the good education to promote their students’ ethical development (Kudo et al., 2013). In the present research, determination and austerity of the teacher cause the students to avoid the teacher and become reluctant toward the course instead of interesting students. Therefore, it seems that, students know flexibility of class environment an important criterion. The findings of a research in Hong Kong showed that teaching style of the teacher in class plays an important role in creating an appropriate educational environment. In addition, applying new approaches that suit the class condition could be effective in flexibility of class atmosphere (Zhang, 2009).

Findings of this study can help identify the elementary contextual aspects that affect humanistic approach in nursing education. This research, performed on the basis nursing students’ lived experience of their relations with academic nurse teachers, describe this experience which seems effective for educational, research and managerial goals. Educational administration on the basis of the findings of this study can be useful in making the evaluation scales of the good teachers in nursing education. Our study was focused on a small number of nursing students in Iran’s cultural context. Therefore, the findings of the current study cannot be generalized to other societies and cultures.

## 5. Conclusion

This study presented greater understanding of experiences obtained by the Iranian nursing students about humanistic approach to nursing education. The findings also show that the humanistic approach to nursing education is a close relation in ethical necessities with effective interaction. In addition, the findings of our research could provide some cases of characteristics of academic nurse teachers in the society of Iran.

## References

[ref1] Borhani F, Alhani F, Mohammadi E, Abbaszadeh A (2010). Professional Ethical Competence in nursing: the role of nursing instructors. Journal of Medical Ethics & History of Medicine.

[ref2] Dunlosky J, Rawson K. A, Marsh E. J, Nathan M. J, Willingham D. T (2013). Improving students’ learning with effective learning techniques: Promising directions from cognitive and educational psychology. Psychological Science in the Public Interest.

[ref3] Elahi N, Alhani F, Ahmadi F (2012). Effective Education: Perceptions and Experiences of Nursing Students. Iranian Journal of Medical Education.

[ref4] Emamzadeh Ghasemi H. S, Rafii F, Farahani M. A, Mohammadi N (2014). Being at peace as an important factor in acquiring teaching competency by Iranian nurse teachers: a qualitative study. Global Journal of Health Science.

[ref5] Grierson A. L (2010). Changing conceptions of effective teacher education: The journey of a novice teacher educator. Studying Teacher Education.

[ref6] Heidarzadeh M, Izadi A, Rahmani A, Zamanzadeh V (2012). Characteristics of efficient clinical teachers: Nursing educators’ and students’ perspectives. Iranian Journal of Medical Education.

[ref7] Hossein K. M, Fatemeh D, Fatemeh O. S, Katri V. J, Tahereh B (2010). Teaching style in clinical nursing education: a qualitative study of Iranian nursing teachers’ experiences. Nurse Education in Practice.

[ref8] Jahanpour F, Sharif F, Salsali M, Kaveh M. H, Williams L. M (2010). Clinical decision-making in senior nursing students in Iran. International Journal of Nursing Practice.

[ref9] Kelly C (2007). Student’s perceptions of effective clinical teaching revisited. Nurse Education Today.

[ref10] Kudo Y, Hayashi S, Yoshimura E, Shibuya A, Aizawa Y (2013). Nursing students’ learning motivation toward technical knowledge and their ethics regarding patients’ rights. The Tohoku Journal of Experimental Medcine.

[ref11] Mazloomy Mahmood Abad S. S, Rahaei Z, Ehrampoush M. H, Soltani T (2010). The characteristics of an expert faculty member based on view points of medical students–Yazd, Iran. Bimonthly Journal of Hormozgan University of Medical Sciences.

[ref12] Ramos F. R, Brehmer L. C, Vargas M. A, Schneider D. G, Drago L. C (2013). Ethics constructed through the process of nurse training: conceptions, spaces and strategies. Revista Latino-Americana de Enfermagem.

[ref13] Sahenk S. S (2010). Characteristics of the headmasters, teachers and students in an effective school. Procedia, Social and Behavioral Sciences.

[ref14] Shahsavari H, ParsaYekta Z, Houser M. L, Ghiyasvandian S (2013). Perceived clinical constraints in the nurse student-instructor interactions: a qualitative study. Nurse Education in Practice.

[ref15] Smedley A, Morey P (2010). Improving learning in the clinical nursing environment: perceptions of senior Australian bachelor of nursing students. Journal of Research in Nursing.

[ref16] Srisawasdi N (2012). Student teachers’ perceptions of computerized laboratory practice for science teaching: A comparative analysis. Procedia-Social and Behavioral Sciences.

[ref17] Streubert H. J, Carpenter D. R (2010). Qualitative research in nursing: Advancing the humanistic imperative.

[ref18] Torrance C, Mansell I, Wilson C (2012). Learning objects?Nurse educators’ views on using patients for student learning: Ethics and consent. Education for Health (Abingdon).

[ref19] Vaismoradi M, Parsa-Yekta Z (2011). Iranian nursing students’ comprehension and experiences regarding evaluation process: a thematic analysis study. Scandinavian Journal of Caring Sciences.

[ref20] Van Manen M (2001). Researching lived Experience: Human Science for An Action Sensitive Pedagogy.

[ref21] Yunus M. M, Osman W. S. W, Ishak N. M (2011). Teacher-student relationship factor affecting motivation and academic achievement in ESL classroom. Procedia, Social and Behavioral Sciences.

[ref22] Zarghami S, Ramezani A (2010). Ethical fundamentals and principles of teacher-student relations on the basis of a comparative analysis and critical study of viewpoints of existence Philosophers. Strategy for Culture.

[ref23] Zhang L. F (2009). From conceptions of effective teachers to styles of teaching: Implications for higher education. Learning and Individual Differences.

